# The comparison of efficacy and safety between transradial and transfemoral approach for chronic total occlusions intervention: a meta-analysis

**DOI:** 10.1038/s41598-022-11763-y

**Published:** 2022-05-09

**Authors:** Wei-Chieh Lee, Po-Jui Wu, Chih-Yuan Fang, Hsiu-Yu Fang, Chiung-Jen Wu, Ping-Yen Liu

**Affiliations:** 1 Institute of Clinical Medicine, College of Medicine, National Cheng Kung University, Tainan, Taiwan, ROC; 2 Division of Cardiology, Department of Internal Medicine, Kaohsiung Chang Gung Memorial Hospital, Chang Gung University College of Medicine, Kaohsiung, Taiwan, ROC; 3 Division of Cardiology, Department of Internal Medicine, Chi Mei Medical Center, Tainan, Taiwan, ROC; 4 Division of Cardiology, Department of Internal Medicine, National Cheng Kung University Hospital, College of Medicine, National Cheng Kung University, 138 Sheng-Li Rd. North District, Tainan, 70403 Taiwan, ROC

**Keywords:** Interventional cardiology, Cardiology, Health care

## Abstract

This meta-analysis compared the outcomes of transradial access (TRA) and transfemoral access (TFA) in chronic total occlusion (CTO) percutaneous coronary intervention (PCI) in recent decades. We searched multiple databases for articles published between January 1, 2015, and December 31, 2020. Six observational studies with 11,736 patients were analyzed. Data included baseline demographics, Japan-chronic total occlusion (J-CTO) score, sheath size, PCI vessel, retrograde method, procedural time, fluoroscopy time, and contrast volume. The more prevalent target CTO vessel was the left coronary artery in the TRA group and the right coronary artery in the TFA group. Higher J-CTO score, longer procedural time, and more contrast volume were seen in the TFA group. In comparison, the TRA group had better procedural success rate (odds ratio (OR), 0.846; 95% confidence interval (CI) 0.749–0.956) and less vascular complications (OR, 0.323; 95% CI 0.203–0.515), but similar retrograde success rate (OR, 0.965; 95% CI 0.382–2.435). In-hospital death (OR, 0.527; 95% CI 0.187–1.489) and major adverse cardiovascular events (OR, 0.729; 95% CI 0.504–1.054) did not differ between the groups. Overall, fewer vascular complications and higher procedural success rates were noted in the TRA CTO PCI population. However, similar retrograde success rates and clinical outcomes were noted between the groups.

## Introduction

The recent European Society of Cardiology guidelines on myocardial revascularization recommend transradial access (TRA) as the standard approach for any percutaneous coronary intervention (PCI), irrespective of clinical presentation, unless there are overriding procedural considerations (recommendation: Class I, and level A)^1^. For diagnostic coronary angiography and percutaneous coronary intervention for coronary artery disease and acute coronary syndrome, TRA reduce short-term net adverse clinical events, cardiac death, all-cause mortality, bleeding, and access site complications, when compared to transfemoral access (TFA)^2,3^. TRA also provides many benefits including lower access-site complications, increased patient comfort, early ambulation, and shorter hospital stay^3–5^.

However, potential disadvantages of TRA include smaller vessel size, vessel spasm, and more techniques for guiding catheter placement^2,6,7^. The main reason for requiring a large guiding system is to be able to use an anchor balloon to help deliver antegrade dissection re-entry equipment or intravascular ultrasound guide puncture of the proximal cap with a microcatheter in situ, or for the need for a debulking device (large-burr rotablator)^8,9^. TFA provides strong backup support, enables the use of multiple equipment combinations, and allows unrestricted use of the trapping technique^10,11^. With the development of Glidesheath and improvement of wires for chronic total occlusion (CTO), the interventionist could try larger sheaths via TRA in recent decades^12^. Therefore, TRA has already become a popular method for CTO PCI, regardless of whether the antegrade or retrograde method was used.

Due to significant advances in specious materials and techniques along with increased operator experience and hybrid strategies, dramatic increment in success rate of CTO PCI in expert hands and low complication rates were reported^13–15^. Most studies comparing between TRA and TFA for CTO PCI were observational studies and did not involve recent improvements^16,17^. Therefore, due to the recent improvements in method and devices for CTO PCI, we focused on recent studies^18–23^ and compared the efficacy and safety between TRA and TFA for CTO PCI.

## Methods

### Search strategies, trial selection, quality assessment, review process, and data extraction

**Figure 1:**
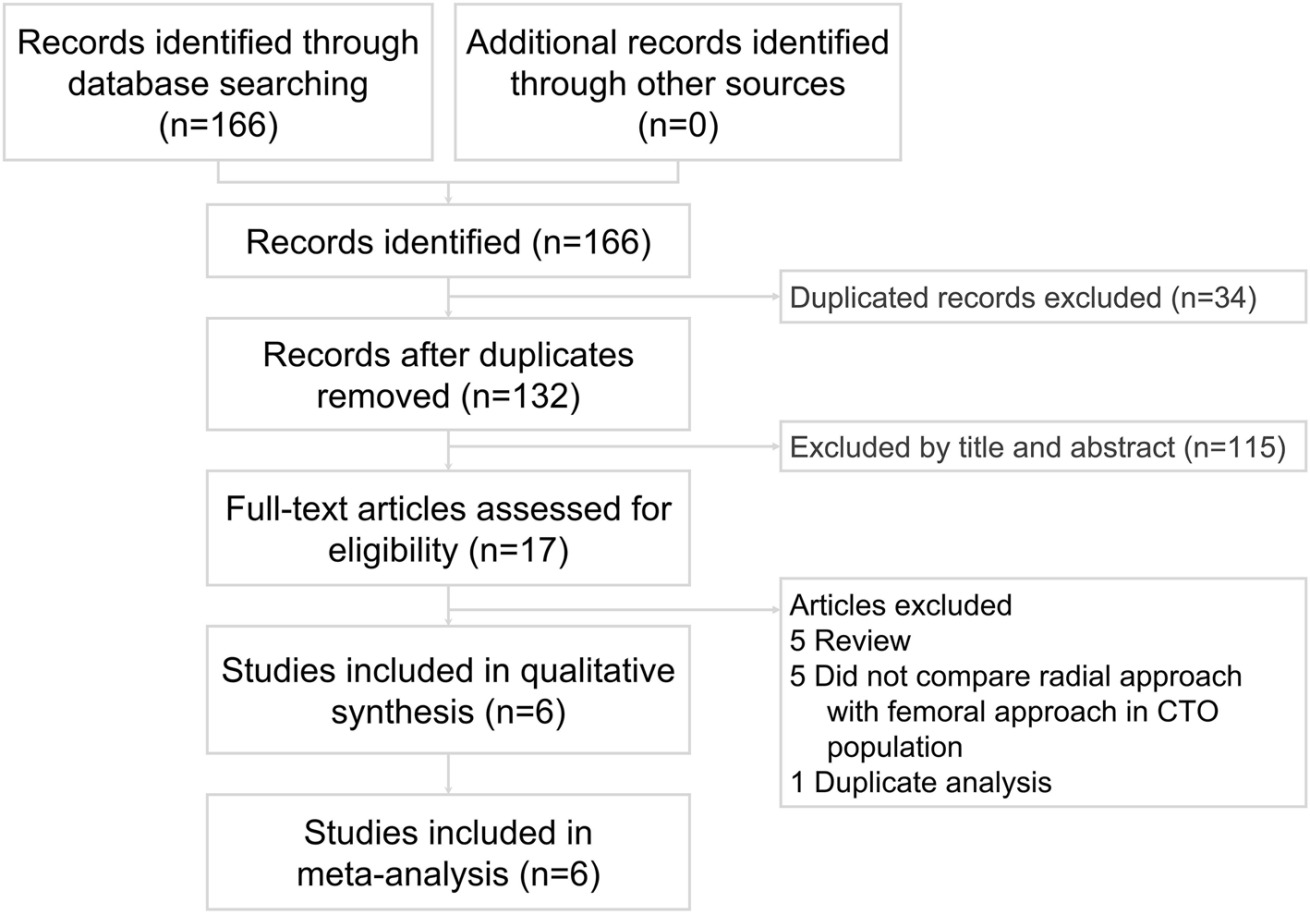
Flowchart of the selection strategy and inclusion and exclusion criteria for this meta-analysis. *CTO* chronic total occlusion.

Figure 1 presents the literature search and screening protocol. A systematic literature searches for published articles between January 1, 2015, and December 31, 2020 in the PubMed, Embase, ProQuest, ScienceDirect, Cochrane Library, ClinicalKey, Web of Science, and ClinicalTrials.gov databases were separately performed by two cardiologists. The searched key terms “chronic total occlusion”, “percutaneous coronary intervention”, “transradial access”, and “transfemoral access” were used. We did not set language restrictions to increase the number of eligible articles. Disagreements were resolved by a third reviewer. Only randomized controlled trials and cohort studies that compared the clinical outcomes of the comparison between TRA and TFA for CTO PCI were included in the present meta-analysis. The inclusion criteria were human studies with a parallel design, with comparison of efficacy and safety between patients with TRA or TFA for CTO PCI. The exclusion criteria included conference abstracts, case reports or series, animal studies, and review articles.

### Definitions

Technical success of six studies was defined as achievement of final flow of target vessel of TIMI grade 3 with at least < 50% residual stenosis of the target CTO lesion at procedure end and was listed in Table 1. Procedural success of two included studies^22,23^ was the same as the definition of clinical trial design principles for CTO therapies^24^ and was defined as technical success plus the absence of an in-hospital major adverse cardiovascular event (MACE) including death, myocardial infarction, or clinically driven target vessel revascularization.

**Table 1:** Characteristics of the 6 included studies.

First author (year)	Patients number (male %)	Age (years)	Study design	Study period	Approach strategy (%)	The definition of approach	The definition of technical/procedural success
Murakami T (2015)^18^	195 (84)	67 ± 11	Single center/retrospective	January 2008 to December 2011	N/A	TRA: single radial; TFA: single femoral or bifemoral	The percentage of diameter stenosis < 50% with TIMI-3 flow (procedural success)
Bakker EJ (2017)^19^	1253 (86)	65 ± 10	Multi-center/RECHARGE registry	January 2014 to October 2015	AWE (79.6%), ADR (15.3%), Retrograde (33.6%)	TRA: single radial or biradial; TFA: single femoral, bifemoral, or combined radial and femoral	The percentage of diameter stenosis < 30% with TIMI-3 flow (technical success)
Kinnaird T (2017)^20^	6480 (82)	64 ± 11	BCIS-NICOR database	January 2006 to December 2013	N/A	TRA: single radial or biradial; TFA: single femoral, bifemoral, or combined radial and femoral	N/A
Tanaka Y (2017)^21^	544 (82)	67 ± 11	Single center/retrospective	January 2005 to December 2014	Retrograde (22.4%)	TRA: single radial or biradial; TFA: single femoral, or bifemoral	The percentage of diameter stenosis < 50% with TIMI-3 flow (technical success)
Huyut MA (2018)^22^	358 (90)	60 ± 10	Single center/retrospective	January 2012 to August 2017	Antegrade (72.3%). Retrograde (27.7%)	TRA: single radial or biradial; TFA: single femoral, or bifemoral	The percentage of diameter stenosis < 30% with TIMI-3 flow (technical success) plus absence of in-hospital complications (procedural success)
Tajti P (2019)^23^	3790 (85)	65 ± 10	Multi-center/PROGRESS CTO registry	May 2012 to July 2018	AWE (83.6%), ADR (29.6%), Retrograde (36.7%)	TRA: single radial or biradial; TFA: single femoral, or bifemoral	The percentage of diameter stenosis < 30% with TIMI-3 flow (technical success) plus absence of in-hospital complications (procedural success)

*AWE* antegrade wire escalation, *ADR* antegrade dissection and re-entry, *TIMI* thrombolysis in myocardial infarction.

### Statistical analysis

All analyses were performed by using Comprehensive Meta-Analysis software, version 3 (Biostat, Englewood, NJ, USA). The frequency of each evaluated outcome was extracted from each study and were showed as cumulative rates. A random effects model was applied to pool individual odds ratios (ORs). The chi‐square test was used to evaluate heterogeneity across trials, (*p* ≤ 0.1 was considered significant). *I*^*2*^ statistics (> 50% was considered high heterogeneity) was employed to examine each outcome. Funnel plots and Egger’s test were used to inspect potential publication bias (*p* ≤ 0.1 was considered significant). p values < 0.05 was defined statistical significance.

## Results

### Characteristics of included studies

The study selection process is displayed in Fig. 1 and six studies met the inclusion criteria. A total of 11,736 participants (mean age of 64.2 ± 10.6 years; 81.7% male) were included. The study design, definition of TRA and TFA, and participants’ characteristics were described in Table 1. The study period, approach method for CTO PCI, and the definition of technical/procedural success were shown in Table 1.

### Patient demographics

Table 2 describes the basic demographics, comorbidities, mean Japan-chronic total occlusion (J-CTO) score, average sheath size, mean procedural time, mean fluoroscopy time, and mean contrast volume of the study patients. The TFA group was older (TRA vs. TFA group, 63.8 ± 10.8 years vs. 64.5 ± 10.5 years, *p* = 0.001). The TFA group had a higher prevalence of diabetes mellitus, hypertension, dyslipidemia, heart failure, prior myocardial infarction, and prior coronary artery bypass grafting than the TRA group. The TFA group had a higher prevalence of right coronary artery involvement than the TRA group (47.4% vs. 54.8%, *p* < 0.001). Higher mean J-CTO scores were noted in the TFA group (2.0 ± 1.2 vs. 2.4 ± 1.3, p < 0.001).

**Table 2:** Patients’ demographics and CTO target vessel.

	TRA	TFA	*p* value
Age (years)	3.8 ± 10.8 (4365)	64.5 ± 10.5 (7371)	< 0.001
Male sex (%)	82.0 (3578)	81.5 (6005)	1.000
Diabetes mellitus (%)	27.7(1209)	33.0 (2430)	< 0.001
Hypertension (%)	69.2 (3019)	72.6 (5350)	< 0.001
Dyslipidemia (%)	70.8 (1145)	83.6(3042)	< 0.001
Heart failure (%)	19.5 (825)	22.1 (1619)	< 0.001
Prior MI (%)	38.8 (1695)	41.5 (3056)	0.004
Prior CABG (%)	11.6 (506)	22.4 (1652)	< 0.001
**CTO target vessel**			
LAD	35.0 (1538)	29.2 (2164)	< 0.001
LCX	24.5 (1078)	18.3 (1356)	< 0.001
RCA	47.4 (2085)	54.8 (4063)	< 0.001
J-CTO score	2.0 ± 1.2 (1636)	2.4 ± 1.3 (3681)	< 0.001
Sheath size (Fr)	6.6 ± 0.5 (1280)	7.3 ± 0.7 (2683)	< 0.001
Procedure time (min)	87.8 ± 36.9 (1053)	106.8 ± 47.5 (3146)	< 0.001
Fluoroscopy time (min)	34.2 ± 22.1 (1647)	40.5 ± 21.9 (3682)	< 0.001
Contrast volume (ml)	244.2 ± 128.2 (1647)	272.4 ± 101.2 (3657)	< 0.001

Data are expressed as mean ± standard deviation or as number (percentage).

*CTO* chronic total occlusion, *TRA* transradial access, *TFA* transfemoral access, *MI* myocardial infarction, *CABG* coronary artery bypass graft, *LAD* left anterior descending artery, *LCX* left circumflex artery, *RCA* right coronary artery, *J-CTO* Japan chronic total occlusion, *Fr* French.

The TFA group had a larger sheath size (6.6 ± 0.5 Fr vs. 7.3 ± 0.7 Fr, *p* < 0.001) than the TRA group. The TRA group had less procedural time (87.8 ± 36.9 min vs. 106.8 ± 47.5 min, *p* < 0.001), less fluoroscopy time (34.2 ± 22.1 min vs. 40.5 ± 21.9 min, *p* < 0.001), and less contrast volume (244.2 ± 128.2 ml vs. 272.4 ± 101.2 ml, *p* < 0.001) than the TFA group.

### Pooled odds ratio of technical/procedural success rate and retrograde success rate of CTO PCI between the TRA and TFA groups

**Figure 2:**
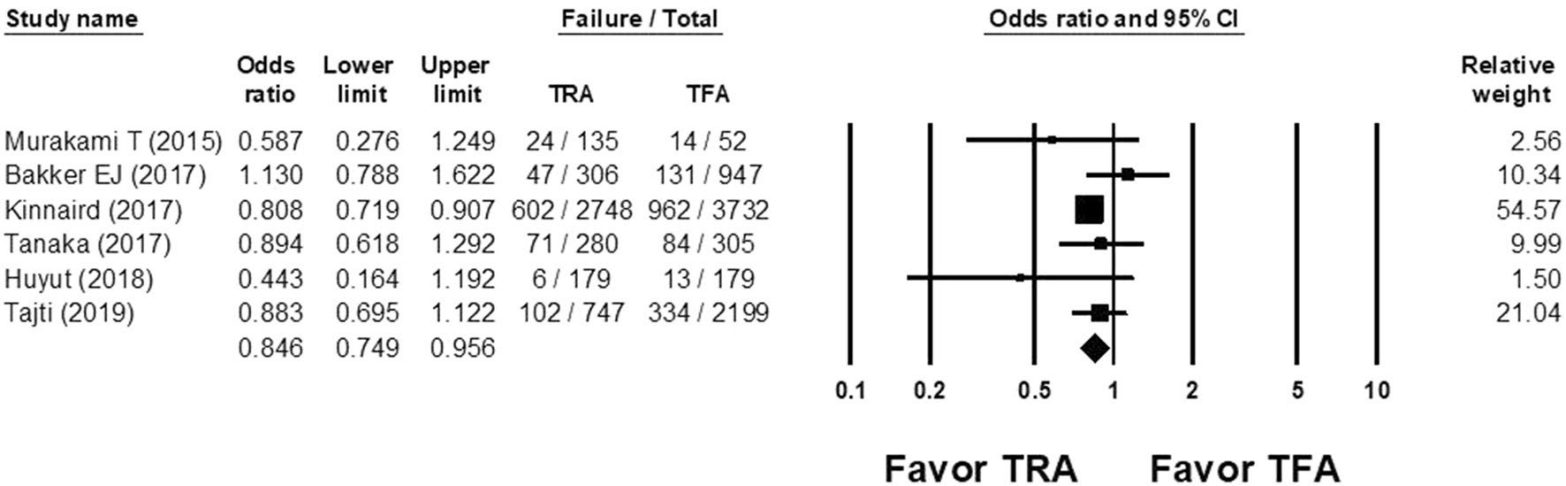
Forest plots of the overall odds ratio (OR) of procedural success rate of chronic total occlusion (CTO) percutaneous coronary intervention (PCI) between the transradial access (TRA) and transfemoral access (TFA) groups from 6 studies. *CI* confidence interval, *TRA* transradial access, *TFA* transfemoral access.

The overall odds ratio (OR) of the technical/procedural success rate of CTO PCI in the TRA group versus the TFA group was 0.846 (95% confidence interval (CI), 0.749–0.956; Fig. 2), with non-significant heterogeneity (Cochran Q, 5.764; *df*, 5; *I*^2^, 13.251%; *p* = 0.330) and non-significant publication bias according to Egger regression (*t*, 0.171; *df*, 4; *p* = 0.873) on inspection of the funnel plot (Supplemental Fig. 1).

**Figure 3:**
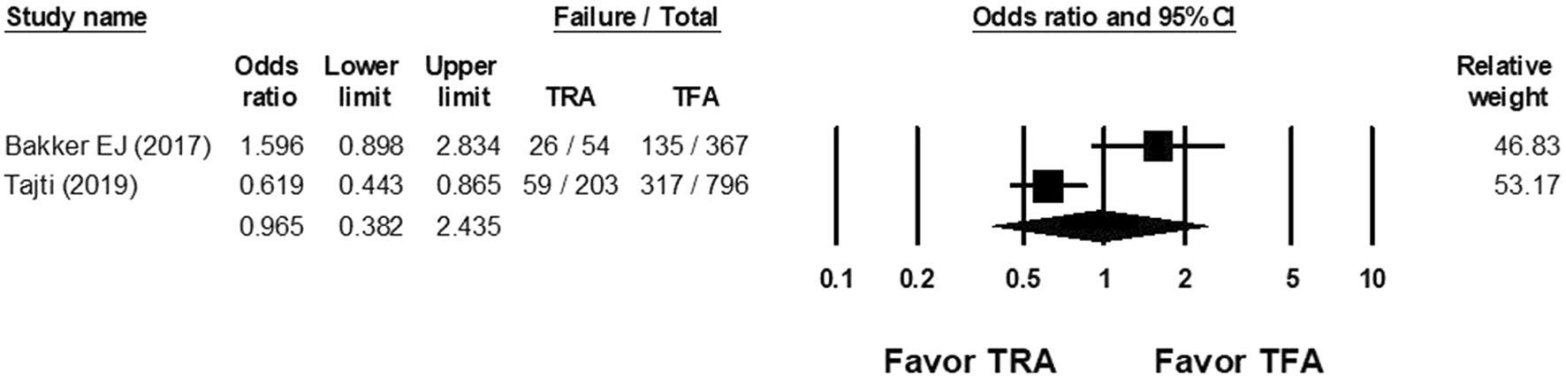
Forest plots of the OR of retrograde success rate of CTO PCI between the TRA and TFA groups from 2 studies.

According to 2 studies, the OR of the retrograde success rate showed that TRA versus TFA for CTO PCI was 0.965 (95% CI 0.382–2.435; Fig. 3), with high heterogeneity (Cochran Q, 7.794; *df*, 1; *I*^2^, 87.169%; *p* = 0.005).

### Pooled odds ratios of vascular complication after CTO PCI

**Figure 4:**
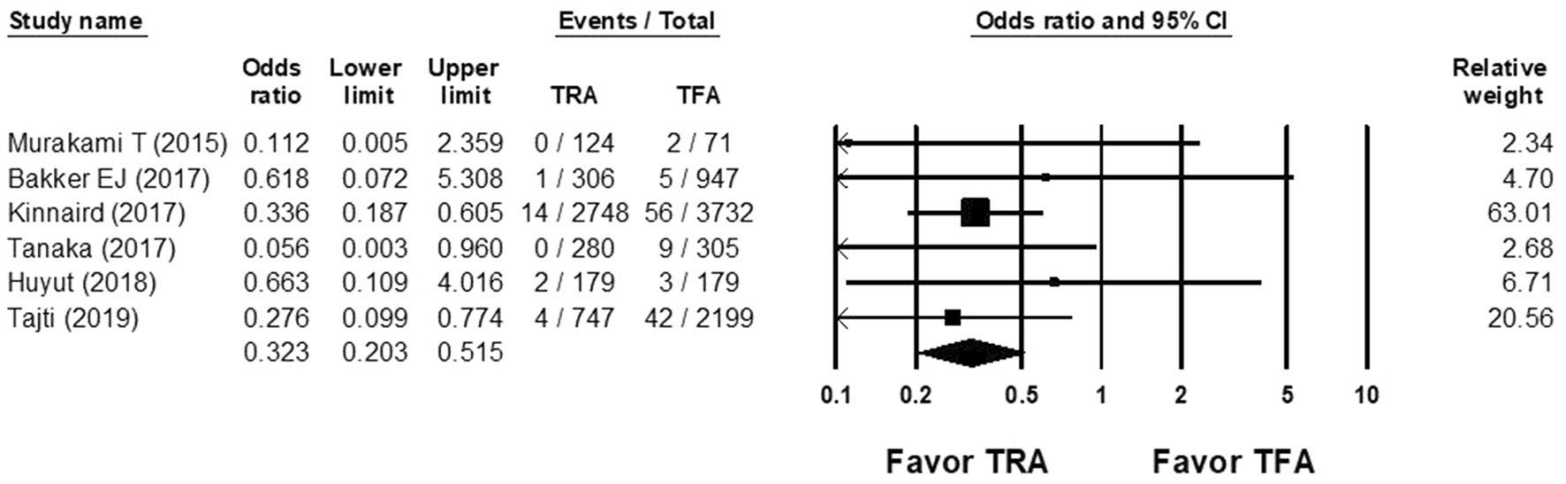
Forest plots of the OR of vascular complication of CTO PCI between the TRA and TFA groups from 6 studies.

The OR of vascular complication of CTO PCI in the TRA group versus the TFA group was 0.323 (95% CI 0.203–0.515; Fig. 4), with non-significant heterogeneity (Cochran Q, 2.997; *df*, 5; *I*^2^, 0%; *p* = 0.700) and non-significant publication bias according to Egger regression (*t*, 0.607; *df*, 4; *p* = 0.577) on inspection of the funnel plot (Supplemental Fig. 2).

### Pooled odds ratios of in-hospital mortality and major adverse cardiovascular event rate after CTO PCI between TRA and TFA groups

**Figure 5:**
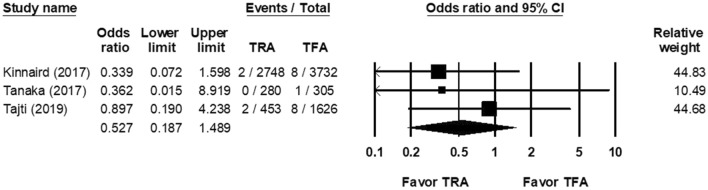
Forest plots of the OR of in-hospital mortality rate of CTO PCI between the TRA and TFA groups from 3 studies.

According to three studies, the OR of in-hospital mortality rate in the TRA group versus the TFA group after CTO PCI was0.527 (95% CI, 0.187–1.489; Fig. 5), with non-significant heterogeneity (Cochran Q, 0.814; *df*, 2; *I*^2^, 0%; *p* = 0.666) and non-significant publication bias according to Egger regression (*t*, 0.277; *df*, 1; *p* = 0.878) on inspection on the funnel plot (Supplemental Fig. 3).

**Figure 6:**
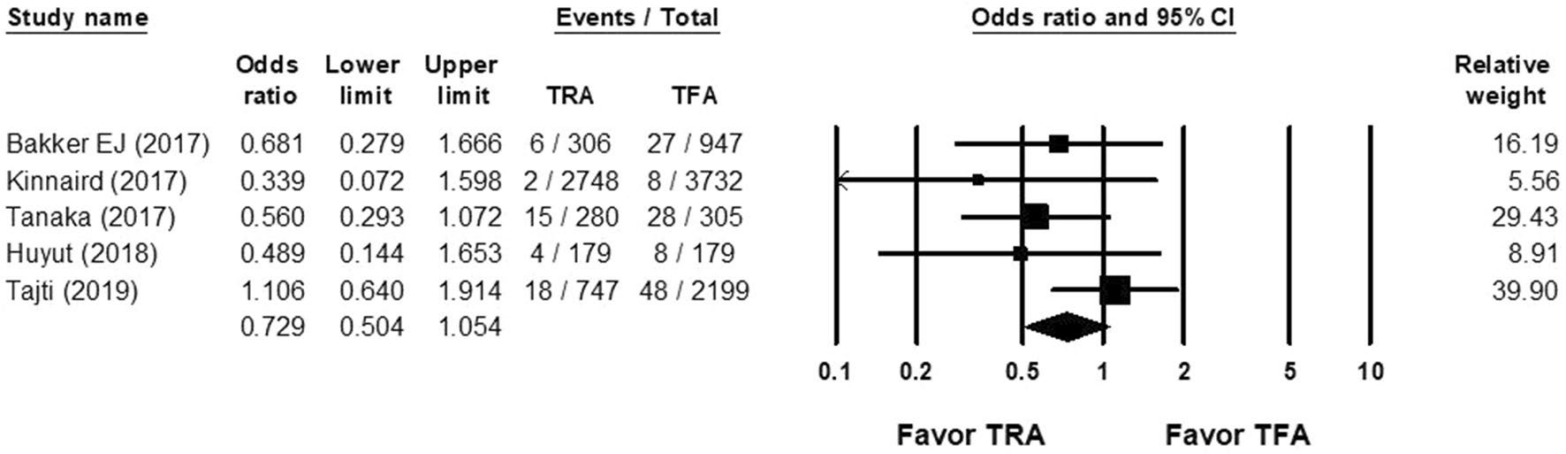
Forest plots of the OR of major adverse cardiovascular event rate of CTO PCI between the TRA and TFA groups from 5 studies.

According to six studies, the OR of MACE rate of the TRA group versus the TFA group after CTO PCI was 0.729 (95% CI, 0.504–1.054; Fig. 6), with non-significant heterogeneity (Cochran Q, 4.229; *df*, 4; *I*^2^, 5.408%; *p* = 0.376) and non-significant publication bias according to Egger regression (*t*, 1.969; *df*, 3; *p* = 0.144) on inspection of the funnel plot (Supplemental Fig. 4).

## Discussion

PCI for CTO lesions in the presence of viable myocardium and improvement of life quality is well accepted by most cardiovascular interventionists^25^. An increasing number of newly developed devices and techniques for CTO intervention and the success rate has increased gradually^26^. The overall procedure success is highly dependent on successful antegrade or retrograde wiring across the occlusion, as well as the support of guiding catheters, guidewires, and other devices. Although the TFA for CTO is still the first choice for most interventional cardiologists, our previous studies have proven the benefits of the TRA in CTO PCI^27^. In the Japan CTO registry, the use of TRA CTO PCI significantly increased over time gradually in recent years^23^. Bedsides, the TFA may be avoided in special situations including abdominal aortic atherosclerotic disease, severe aortoiliac disease, and previous iliofemoral bypass graft placement^28^.

The important limitation of radial artery is relatively small size. The radial artery is smaller than the femoral artery and may not be suitable for larger sheaths and guiding catheters. On routine diagnostic coronary angiography and PCI for acute coronary syndrome (ACS), TRA is a valuable alternative to TFA associated with a reduction in vascular complications^29,30^. However, routine diagnostic coronary angiography and ACS PCI is not like CTO PCI. Also, complex techniques including the anchor balloon technique, antegrade dissection re-entry technique (ADR), or intravascular ultrasound (IVUS) guide puncture, may require larger bore catheters. After the development of Glidesheath (Terumo, Japan), interventionists could try larger artery sheaths via radial arteries or even distal radial arteries for complex PCI^12^. A high success and low complication rate of the hybrid approach to CTO crossing was reported^13^. The introduction of using enabling strategies including antegrade or retrograde wire escalation, and dissection reentry techniques also brought an increasing success rate of CTO PCI^14^. Therefore, we need to evaluate the efficacy of TRA CTO PCI in recent decades.

This meta-analysis showed a better procedural success rate in TRA CTO PCI than in TFA CTO PCI. However, more comorbidities and higher mean J-CTO scores were noted in the TFA group, which also influenced the procedural success rate. The retrograde success rate was similar between the TRA and TFA groups. Therefore, TRA provides a better procedural success rate and does not improve the retrograde success rate. In addition, less procedural time (87.8 ± 36.9 min vs. 106.8 ± 47.5 min, *p* < 0.001), less fluoroscopy time (34.2 ± 22.1 min vs. 40.5 ± 21.9 min, *p* < 0.001), and less contrast volume (244.2 ± 128.2 ml vs. 272.4 ± 101.2 ml, *p* < 0.001) were seen in the TRA group. In the enrolled studies, two studies reported the incidence of contrast-induced nephropathy, which did not differ between the TRA and TFA groups^21,22^. Shorter procedural time, fluoroscopy time, and less contrast exposure may contribute to fewer procedural complications.

In our study, fewer vascular complications (OR: 0.323; 95% CI 0.203–0.515) were observed in TRA CTO PCI. This may bring about a net clinical benefit by decreasing ischemic events due to cessation of antiplatelet and antithrombotic agents if bleeding, and the adverse effects of blood transfusion^31^. Because TRA reduces vascular access-site complications in patients undergoing PCI for simple or complex procedures, mortality and ischemic events may also be reduced by TRA when compared with TFA. However, a more prevalence of multiple comorbidities and higher J-CTO score also effect the results of vascular complications, associated MACE and mortality. The complexity of baseline characteristics may let interventionists to choose TFA approach for CTO PCI. Therefore, the results of observational studies existed selection bias. Kinnaird et al. reported higher short-term and one-year mortality rates in patients with access-site complications^20^. Tajti et al. reported no significant difference in in-hospital MACE between the bilateral TRA and bilateral TFA groups^23^. In our study, for the incidence of in-hospital mortality (OR: 0.527; 95% CI 0.187–1.489) and MACE (OR: 0.729; 95% CI 0.504–1.054), TRA CTO PCI showed a non-significant trend when compared with TFA CTO PCI. In addition, a recent randomized study reported TRA is associated with a significant reduction in clinically relevant access-site bleeding or vascular complications, without affecting procedural success when compared with TFA in patients undergoing PCI of complex coronary lesions (≥ 50% CTO PCI) with large-bore access^32^. In our study, higher procedural success rates were noted in the TRA CTO PCI may be contributed by better baseline characteristics and lower J-CTO score, but fewer vascular complications may be associated with better vascular condition and the benefit of TRA.

## Limitations

This study has several limitations. First, all the studies were observational cohort studies and not all studies provide detailed information about the procedure about CTO PCI. Because of observational studies, selection bias could not be excluded totally, and the difference of baseline characteristics could influence the results. Second, we could not decrease the bias from the difference of baseline characteristics. Third, the operator’s experience and CTO PCI volume are not available in all enrolled studies, but all data was from experienced centers or registry of muti-center. Only one study mentioned the operators’ experience as a minimum of 25 hybrid procedures and certification for the controlled antegrade dissection re-entry technique^19^. Third, the definition of technical/procedural success was different in the included studies. However, a total of 11,736 participants were enrolled from 6 studies. The present study still provides some important findings on the outcomes of TRA CTO PCI after the improvement of the CTO technique and devices in recent decades.

## Conclusion

Due to the improvement of the CTO technique, fewer vascular complications and higher procedural success rates were noted in the TRA CTO PCI population. However, similar retrograde success rates and clinical outcomes were noted between the groups. More complex comorbidities and higher J-CTO scores still influenced outcomes.

## Supplementary Information

Supplementary Figures.
