# Supplementary material for: The comparison of efficacy and safety between transradial and transfemoral approach for chronic total occlusions intervention: a meta-analysis
Source: Sci Rep. 2022 May 9;12:7591. doi: 10.1038/s41598-022-11763-y (PMC9085849; doi:10.1038/s41598-022-11763-y)
Supplement: Supplementary file 1 — Supplementary Figures. [file 41598_2022_11763_MOESM1_ESM.pdf]

Supplemental Figures and Figure Legends

Figure 1.

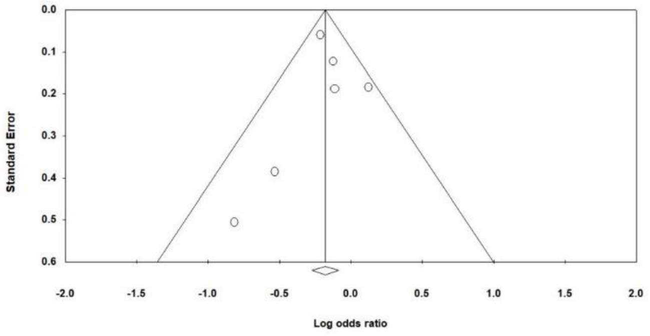

The funnel plot showed non-significant publication bias according to Egger regression (*t*, 0.171; *df*, 4; *p* = 0.873).

Figure 2.

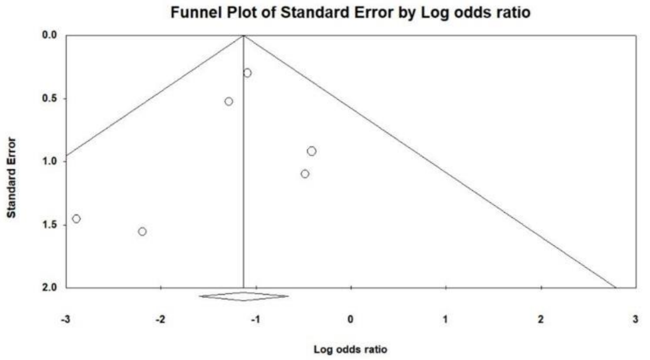

The funnel plot showed non-significant publication bias according to Egger regression (*t*, 0.607; *df*, 4; *p* = 0.577).

Figure 3.

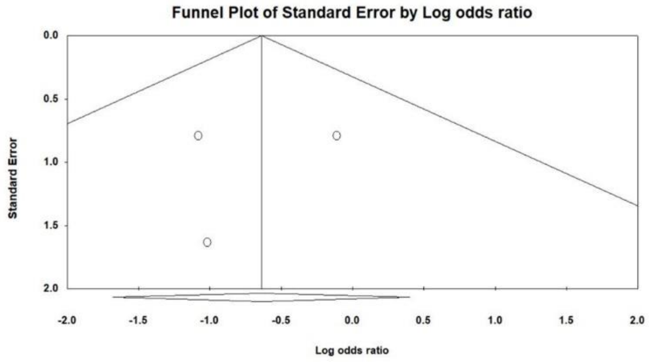

The funnel plot showed non-significant publication bias according to Egger regression (*t*, 0.277; *df*, 1; *p* = 0.878).

Figure 4.

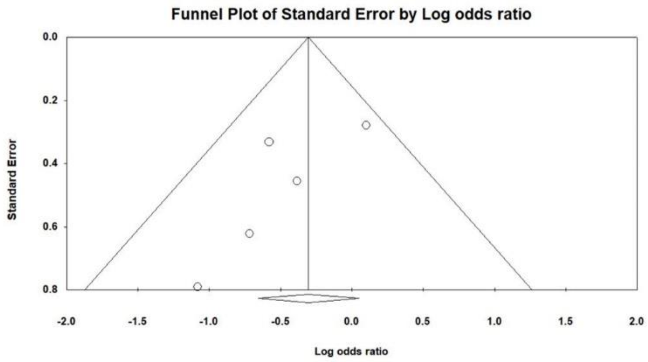

The funnel plot showed non-significant publication bias according to Egger regression (*t*, 1.969; *df*, 3; *p* = 0.144).
